# Aspirin suppresses chemoresistance and enhances antitumor activity of 5-Fu in 5-Fu-resistant colorectal cancer by abolishing 5-Fu-induced NF-κB activation

**DOI:** 10.1038/s41598-019-53276-1

**Published:** 2019-11-15

**Authors:** Jinbo Fu, Yiming Xu, Yushan Yang, Yun Liu, Lulu Ma, Yiyao Zhang

**Affiliations:** 1 Departments of Gastrointestinal Surgery, Zhongshan Hospital of Xiamen University, Xiamen, 361000 China; 2 General Surgery, Zhongshan Hospital of Xiamen University, Xiamen, 361000 China; 3 Gastrointestinal Oncology Center of Xiamen University, Xiamen, 361000 China; 4 Medical College of Xiamen University, Xiamen, 361000 China

**Keywords:** Translational research, Cancer therapeutic resistance

## Abstract

Chemoresistance to 5-fluorouracil (5-Fu)-based chemotherapy is a leading obstacle in achieving effective treatment for colorectal cancer (CRC). Typically, NF-κB activation induced by the chemotherapeutics themselves is an important cause resulting in chemoresistance. Specifically, NF-κB activation can inhibit tumor cell apoptosis and induce chemoresistance. Drugs that can prevent NF-κB activation induced by chemotherapeutics are urgently needed to overcome chemoresistance. Obviously, aspirin is one of these agents, which has been demonstrated to possess antitumor activities and as an inhibitor of NF-κB. The current study aimed to investigate whether aspirin was able to overcome the chemoresistance to 5-Fu in CRC, together with the potential synergistic mechanisms. Our results suggested that aspirin remarkably potentiated the inhibitory effect of 5-Fu on the growth and invasion of resistant cells *in vitro*. *In vivo*, aspirin markedly enhanced the antitumor activity of 5-Fu in suppressing tumor growth and metastasis, and down-regulating the expression of NF-κB-regulated genes in the 5-Fu-resistant cells. Obviously, aspirin completely eradicated the 5-Fu-induced NF-κB activation, without inducing pronounced adverse effects. Taken together, findings in this study suggest that aspirin can reverse chemoresistance and potentiate the antitumor effect of 5-Fu, which is achieved through abolishing the 5-Fu-induced NF-κB activation, suggesting that aspirin may be a promising adjuvant therapeutic agent for CRC.

## Introduction

Colorectal cancer (CRC), one of the most common malignancies, accounts for the second leading cause of cancer-related deaths in the USA^1^. An increasing number of new agents have emerged, nonetheless, the 5-fluorouracil (5-Fu)-based combined chemotherapy remains the preferred choice for CRC patients. However, the overall response rate of CRC to 5-Fu is <15% due to the intrinsic or acquired chemoresistance in exposure to 5-Fu^2^. The presence of chemoresistance has prompted 50% patients to develop liver metastasis during disease progression eventually^3,4^. Although treatment with 5-Fu in combination with other chemotherapeutics can improve the response rate, it will also remarkably lead to severe adverse effects (AEs)^4^. Therefore, it is urgently needed to develop the less toxic agents to enhance the sensitivity of CRC cells to 5-Fu or to prevent the resistance to 5-Fu.

Aspirin has been extensively used clinically as an agent to prevent cardiovascular events. Moreover, both epidemiological and clinical studies have demonstrated that aspirin can potentially reduce the incidence and metastasis of CRC^5–8^. One recent study demonstrates that aspirin probably improves the clinical outcomes of metastatic CRC patients receiving capecitabine, a derivative of 5-Fu, after the failure of standard chemotherapy^9^. Such results suggested that aspirin potentially boosted the chemosensitivity of CRC to 5-Fu-based chemotherapy. The mechanisms underlying the antitumor activity of aspirin remain hypothetical and complex, yet it may be one of the critical mechanisms that the antitumor effect of aspirin on CRC targets nuclear factor- κB (NF-κB), the transcription factor^10,11^.

NF-κB has been reported in numerous studies to exert an important role in tumorigenesis, tumor chemoresistance and metastasis, which is achieved through mediating the transcription of a serial of genes, including pro-proliferative gene cyclin D1, anti-apoptotic gene Bcl-2 and Survivin, and the angiogenetic gene VEGF^12–16^. The ability to induce NF-κB translocation and activate its target genes is one common feature of chemotherapeutics, which is the main reason that tumor can acquire chemoresistance to the anticancer agents^10^. Thus, inhibiting NF-κB activation can reverse the chemoresistance and enhance the efficacy of anticancer agents in numerous cancer types, including CRC^10,17–20^. Recently, NF-κB has been identified as a crucial regulator of epithelial-mesenchymal transition (EMT) in CRC^21^, which is essential for tumor metastasis and chemoresistance^22,23^. NF-κB over-expression is also closely associated with advanced CRC, which may predict a worse overall survival^24^. Consequently, the NF-κB pathway represents a potentially promising therapeutic target, and NF-κB inhibitors can be used to overcome the chemoresistance of CRC to chemotherapeutics. Although there are overwhelming proofs that aspirin can prevent CRC, very few studies have focused on the underlying mechanism of action by which aspirin improves the sensitivity of chemoresistant CRC to chemotherapeutics.

In this study, the innate or acquired resistance of 5-Fu in CRC cells was mimicked to assess whether aspirin reversed the resistance of CRC cells to 5-Fu and to further investigate the underlying synergic mechanisms. Our results suggested that aspirin, as a sensitizer, reversed the chemoresistance of CRC cells to 5-Fu and enhanced the antitumor effects of 5-Fu both *in vivo* and *in vitro*, without inducing additional toxicity, and such effects were achieved by abolishing the 5-Fu-induced NF-κB activation.

## Materials and Methods

### Established human CRC cell lines and reagents

The established human CRC cell lines SW480 and SW620 were obtained from American Type Culture Collection (ATCC) and cultured in the Dulbecco’s modified eagle medium (DMEM) supplemented with 10% fetal bovine serum (FBS). Then, cells were incubated in a humidified incubator under 37 °C and 5% CO2 conditions. Additionally, the 5-Fu resistant cell lines SW480-FU and SW620-FU were derived through gradually increasing 5-Fu concentrations in SW620 and SW480 cell lines, as described previously^17^. After sequential 5-Fu treatments from 10 μM to 100 μM for 3 months, both SW480-FU and SW620-FU cell lines were selected from the parental cell lines, which were totally resistant to 100μM 5-Fu. Moreover, mycoplasma tests were also performed monthly to ensure that the cultures were mycoplasma-negative. Aspirin and 5-Fu were purchased from the Pharmacy of Zhongshan Hospital of Xiamen University and Sigma, respectively. Notably, aspirin and 5-Fu powder were dissolved in dimethyl sulfoxide (DMSO) and preserved in aliquots at −20 °C. The final solvent concentrations in the medium were <0.1%.

### Cell viability assay

Cell viability was determined according to the 3-(4,5-dimethylthiazol-2-yl)-2, 5-diphenyltetrazolium bromide (MTT) assay, as described in a previous study^17^.

### Detection of apoptosis

Alexa fluoresceinisothiocyanate-conjugated Annexin V (BD Bioscience, San Jose, CA, USA) was employed to determine the number of apoptotic cells in accordance with manufacturer protocols, as described previously^17^.

### Western blotting

Western blotting was performed as previously described^17^. In brief, the whole-cell or nuclear extracts from CRC cells and tumor samples were prepared according to a standard protocol. Then, the samples were subjected to 10% sodium dodecyl sulfate-polyacrylamide gel electrophoresis (SDS-PAGE) for separation. Afterwards, the separated samples were transferred onto the polyvinylidene difluoride (PVDF) membranes (Millipore, CA). Later, the membranes were blocked with 5% milk and incubated with the following primary antibodies, including p65 and p-p65 (Santa Cruz Biotechnology, CA, USA), cyclin D1, VEGF, Bcl-2, Survivin, Ki67 (Abcam, Cambridge, UK), cleaved Caspase-3, p-IKK-β, and p-IKK−α (Cell Signaling, MA, USA) antibodies, at 4 °C overnight, followed by incubation with secondary antibodies. Finally, the proteins were visualized using the ECL detection reagent.

### Electrophoretic mobility shift assay (EMSA)

EMSAs were carried out as described previously^25^. Briefly, the nuclear extracts from CRC cells and tumor samples were incubated with the radiolabeled DNA probes containing a consensus κB site for 30 min at room temperature. Later, the formed DNA-protein complexes were separated from the free oligonucleotides on the native 4% polyacrylamide gel and visualized by means of autoradiography.

### Transfection of small interfering RNA

Cells in 6-well plates or 96-well plates were grown to 50% confluence and transfected with p65 siRNA (Dharmacon Res Inc.) or with a siRNA nonspecific control (Ambion, Austin, TX cat# 4611). Cells were incubated for 48 h and p65 protein expression was then confirmed by Western blotting. Alternatively, cells were treated with 5-FU or aspirin or both agents together for another 72 h. Then, the viability of siRNA-transfected cells under different treatments were detected with MTT.

### Migration and invasion assays

The migration and invasion capacities of cells were evaluated through Transwell assay. In brief, the Transwell chambers were coated with or without 100 mL diluted matrigel (Costar, Cambridge, MA). Then, cells were suspended within 100 µl serum-free medium and seeded into the upper Transwell chamber at a density of 2 × 10^5^ cells/ml, whereas the lower Transwell chamber was filled with medium containing 10% FBS. After treatment for 48 h in each group, cells on the upper chamber surface that had not migrated were carefully removed with a cotton swab. Afterwards, the remaining cells were fixed with cold 95% ethanol, and stained with 0.1% crystal violet. The experiments were repeated in triplicate wells, and the number of migrating cells was quantified by counting the cells in five random fields per filter under the microscope.

### Xenografts in nude mice

All animal experiments and procedures were carried out in accordance with the protocol approved by the Animal Care and Ethics Committee of Xiamen University. To confirm the above results in animals, both SW620 and SW620-FU cell lines were selected to form the xenografts in nude mice. Briefly, SW620 (1 × 10^6^) or SW620-FU (5 × 10^5^) cells were suspended within 100 μL matrigel diluted in DMEM (1:1), which were then injected into the left rear flank of the 6-to-8-week-old female Balb/c nude mice (day 0). After 3 days of implantation when tumors had reached a palpable size, mice bearing tumors from SW620 cells were randomly divided into 4 groups (n = 7 in each group) and treated as follows: (a) control group (100 μL of 0.9% saline, oral gavage, daily); (b) aspirin group (200 mg/kg aspirin dissolved in saline, oral gavage, daily); (c) 5-Fu group (20 mg/kg, intraperitoneal injection, twice weekly); and (d) 5-Fu combined with aspirin group (20 mg/kg 5-Fu, intraperitoneal injection, twice weekly; and 200 mg/kg aspirin dissolved in saline, oral gavage, daily). Similarly, 40 mice bearing tumors from SW620-FU cells were also randomly divided into 8 groups (n = 5 in each group) and treated as follows: (a) control group (100 ml of 0.9% saline, oral gavage, daily); (b) low-dose aspirin group (100 mg/kg aspirin dissolved in 0.9% saline, oral gavage, daily); (c) moderate-dose aspirin group (200 mg/kg aspirin dissolved in saline, oral gavage, daily); (d) high-dose aspirin group (300 mg/kg aspirin dissolved in 0.9% saline, oral gavage, daily); (e) 5-Fu group (25 mg/kg, intraperitoneal injection, twice weekly; 100 μl of 0.9% saline, oral gavage, daily); (f) 5-Fu combined with low-dose aspirin group (25 mg/kg 5-Fu, intraperitoneal injection, twice weekly; 100 mg/kg aspirin dissolved in saline, oral gavage, daily); (g) 5-Fu combined with moderate-dose aspirin group (5-Fu, 25 mg/kg, intraperitoneal injection, twice weekly; 200 mg/kg aspirin dissolved in saline, oral gavage, daily); and (h) 5-Fu combined with high-dose aspirin group (25 mg/k 5-Fu, intraperitoneal injection, twice weekly; 300 mg/kg aspirin dissolved in saline, oral gavage, daily). The animals were euthanized on day 23 and the tumors were excised. The tumor volume was calculated for three times a week according to the following formula: V = 1/2 (length × width2). Moreover, the tumor tissues were immediately frozen in liquid nitrogen and preserved at −80 °C.

### Splenic vein metastasis assay in mice

To evaluate liver and spleen metastases, SW620-FU cells (5 × 10^5^) were injected into the 6-to-8-week-old Balb/c nude mice through the splenic vein. Typically, 28 mice were divided into 4 groups, with 7 in each group, and animals were anaesthetized after 5 weeks to assess the liver and spleen metastases.

### Statistical analysis

The significance of data was analyzed using the Student’s *t*-test for parametric data and the Mann-Whitney test with Bonferroni corrections for non-parametric data. A difference of *P* < 0.05 was considered as statistically significant (*P < 0.05, **P < 0.01).

## Results

### Aspirin enhanced the cytotoxicity of 5-Fu in chemoresistant CRC cells

**Figure 1:**
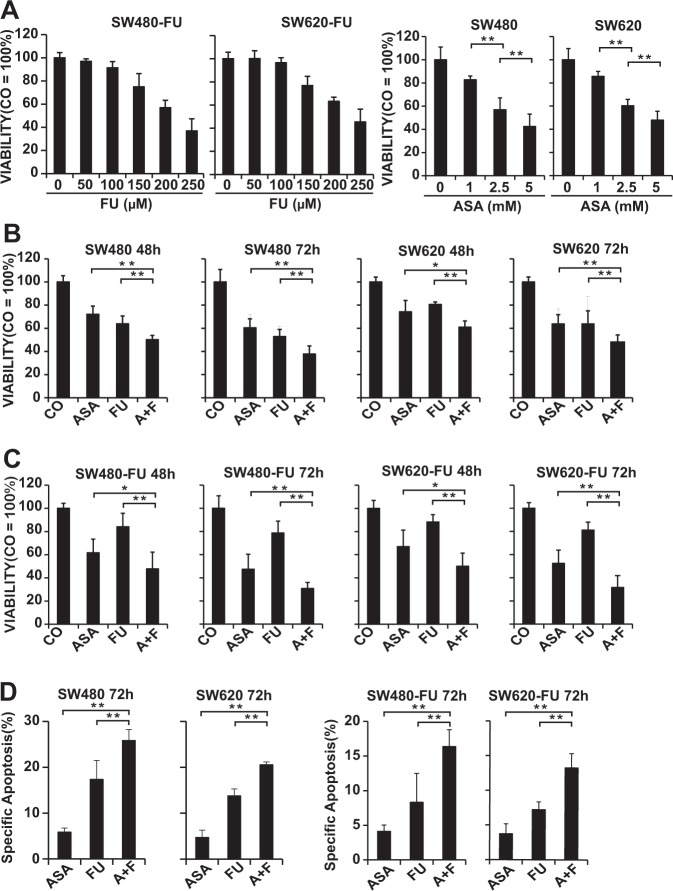
Aspirin overcomes the resistance to 5-Fu and enhances the sensitivity to 5-Fu in CRC cells. **(A**) The established 5-Fu resistant CRC cell lines SW620-FU and SW480-FU were treated with indicated concentrations of aspirin (ASA) or 5-Fu (FU), respectively, and the cell viability was measured at 72 h through MTT assay. **(B)** SW480 and SW620 were treated by 2.5 mM ASA, 20 μM FU, or both of the two drugs (A + F), respectively, and the cell viability was measured at 48 h and 72 h, separately, by MTT assay. **(C)** SW480-FU and SW620-FU were treated by 2.5 mM ASA, 150 μM FU, or both of the two drugs (A + F), respectively, and the cell viability was measured at 48 h and 72 h, separately, by MTT assay. **(D)** CRC cells were treated as described above, and apoptosis was measured at 72 h later by FACS analysis. The data are presented as means ± SD (**P < 0.01, *P < 0.05).

Both SW480 and SW620 cells had innate resistance and derived from the same patient, among which, SW480 cells were obtained from the primary lesion, whereas SW620 cells were acquired from the metastases, but they showed distinctly different sensitivities to 5-Fu. For instance, the IC50 values of 5-Fu based on our MTT data were 18.8 μM for SW480 cells and 29.1 μM for SW620 cells at 72 h (Supplementary Fig. 1). To induce the acquired resistance to 5-Fu, both SW480 and SW620 cells were incubated with 5-Fu at increasing concentrations for several months, so as to obtain the 5-Fu resistant SW480-FU (IC 50: 216.9 μM at 72 h) and SW620-FU (IC 50: 231.4 μM at 72 h) cell lines (Fig. 1A). According to our MTT results, the cell viability was only partly inhibited when treated with 150 μM 5-Fu in SW480-FU and SW620-FU cells, so 150 μM 5-Fu was selected as the concentration for use in subsequent experiments.

To examine whether there was cross-resistance between aspirin and 5-Fu, the effects of aspirin on the proliferation of 5-Fu-resistant SW480-FU and SW620-FU cells, as well as the parental SW480 and SW620 cells, were detected by MTT assay. The results showed that aspirin reduced cell viability in a dose-dependent manner, and no obvious difference was observed between 5-Fu-resistant cells and their parental cells, suggesting no cross-resistance between aspirin and 5-Fu (Fig. 1A–C).

To assess whether aspirin boosted the sensitivity of chemoresistant CRC cells to 5-Fu, the 5-Fu-resistant cells and parental cells were treated with aspirin, 5-Fu alone, or the combination of these two drugs, and cell viability was assessed at 48 h and 72 h, respectively, by MTT assay. As expected, 5-Fu had the minimal effect on both 5-Fu-resistant cells and the parental cells. However, the combined treatment had exerted the greatest effect on inhibiting the proliferation of all the four cell lines in a time-dependent manner, especially in SW480-FU and SW620-FU cells, revealing that the combined treatment effectively overcame the chemoresistance of CRC to 5-Fu (Fig. 1B,C)

Next, cell apoptosis was detected through flow cytometry. Compared with aspirin, aspirin combined with 5-Fu had a more pronounced effect on inducing apoptosis relative to that of 5-Fu alone (Fig. 1D).

Taken together, these results demonstrated that aspirin enhanced the sensitivity of CRC cells to 5-Fu and increased the cytotoxicity of 5-Fu in chemoresistant CRC cells.

### Aspirin inhibited restored the sensitivity of chemoresistant CRC cells to 5-Fu by abolishing 5-Fu-induced NF-κB activation *in vitro*

**Figure 2:**
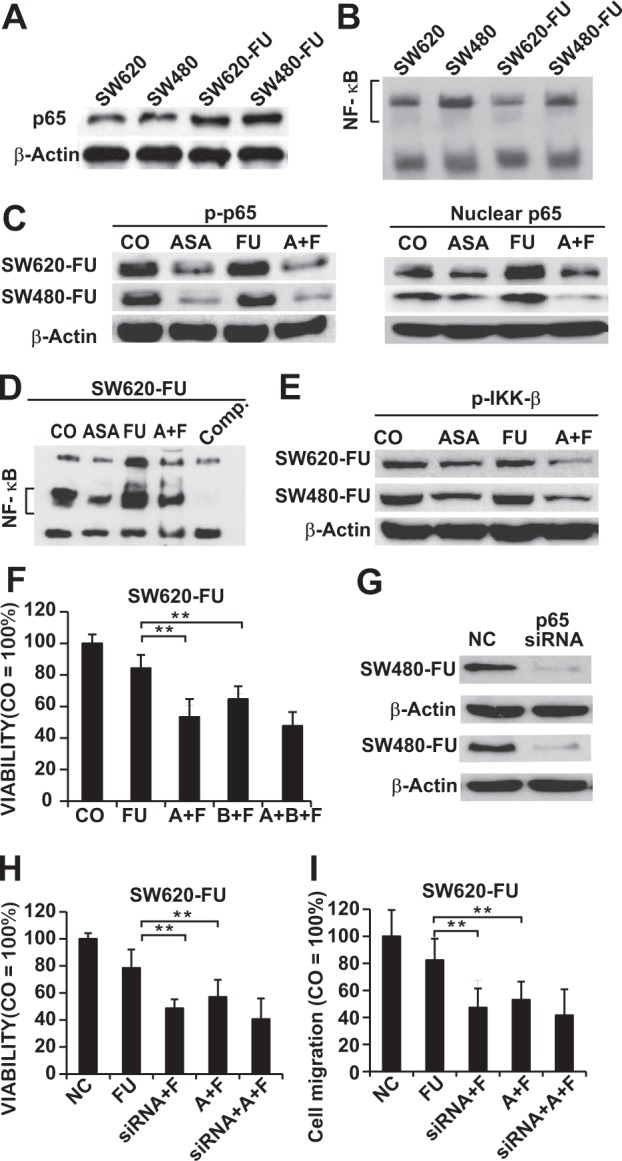
Aspirin enhances the anti-tumor effect of 5-FU by inhibiting the 5-Fu-induced abnormal NF-κB abnormal in resistant CRC cells. (**A)** Nuclear protein was extracted and the basic expression levels of p65 in SW620, SW480, SW620-FU and SW480-FU were determined by Western blotting analysis. **(B)** NF-κB was detected by DNA binding in SW620, SW480, SW620-FU, and SW480-FU through the electrophoretic mobility shift assay (EMSA). **(C)** SW620-FU and SW480-FU cells were treated with 2.5 mM ASA, 150 μM (FU), or both of the two drugs (A + F), respectively, and the expression of nuclear p65 and p-p65 was measured at 48 h through Western blotting analysis. β-Actin serves as a control. **(D)** The NF-κB activity in SW620-FU cells is analyzed by EMSA after 6 h of treatment as described above. Comp: a 10-fold excess of unlabeled oligonucleotide is added to the untreated cells for control DNA-binding reaction, followed by incubation with the biotin-labeled oligonucleotide probe for NF-κB. **(E)** The expression of IKK-β was measured at 48 h through Western blotting analysis as described above. **(F)** SW620-FU cells were treated with 150 μM FU alone, the double combination of 2.5 mM ASA plus 150 μM FU(A + F) or 10 μM Bay11-7082 (A + B), or triple agents combination (A + B + F), respectively, and the cellular viability was measured 72 h later with an MTT assay. **(G)** Western blotting was conducted 48 h after treatment with nonspecific control siRNA (NC) or p65 siRNA. **(H)** Cells were transfected with p65 siRNA for 24 h. Then, cells treated with 5-FU or aspirin or both agents together for another 72 h and the viability of cells were detected with MTT. **(I)**The indicated cells transfected with or without p65-siRNA for 24 h were treated with 5-FU or aspirin or both agents together for 24 hours, and the invasion cells were evaluated using the Transwell chambers coated with Matrigel.

To further examine the underlying molecular mechanism for the chemoresistance to 5-Fu, the activation of the NF-κB signaling in 5-Fu,resistant SW480-FU and SW620-FU cells and their parental cells SW480 and SW620 was investigated. Our results suggested that the expression of p65, a key subunit of NF-κB, was dramatically up-regulated in 5-Fu resistant cells compared with that in their parental cells (Fig. 2A). To further verify the above results, the activity of NF-κB was tested by EMSA, and the results also indicated that the 5-Fu resistant cells showed higher basic NF-κB activity than that in their parental cells (Fig. 2B).

Subsequently, Western blotting and EMSA were carried out to investigate whether aspirin sensitized the chemoresistant CRC cells through affecting the NF-κB pathway. Unexpectedly, our results demonstrated that the 5-Fu-induced the expression of NF-κB (p65, p-p65), and that aspirin treatment not only evidently reduced the expression of NF-κB, but also totally abolished the 5-Fu-induced expression of NF-κB in both 5-Fu resistant cell lines (Fig. 2C). Similarly, the 5-Fu-induced NF-kB DNA binding activity was completely prevented upon aspirin treatment in both chemoresistant CRC cell lines (Fig. 2D).

One of the crucial steps to activate the NF-kB pathway was the phosphorylation by IKK-β and IKK-β and IKK-α; as a result, we investigated whether aspirin inhibited the activation of the NF-kB pathway through suppressing the expression of p-IKK-β and p-IKK-α. Our findings revealed that aspirin ectopically reduced the expression of p-IKK-β but not that of p-IKK-α (data not shown) in the chemoresistant CRC cells (Fig. 2E). Moreover, aspirin also suppressed the 5-Fu-induced expression of p-IKK-β.

In order to further confirm whether enhanced the cytotoxicity of 5-Fu in chemoresistant CRC cells on depended on the NF-kB pathway, BAY11-7082, a NF-κB inhibitor, was used to block NF-κB signaling. According to our results, BAY11-7082, which was almost as strong as aspirin, significantly enhanced the sensitivity of SW620-FU cells to 5-FU, with drastically increased growth-inhibitory effects after treatment. However, the triple treatment with 5-Fu plus aspirin and BAY11-7082 did not show stronger growth-inhibitory effects than that in the combined treatment with 5-Fu plus aspirin (Fig. 2F, Supplementary Fig. 2A). Similarly, aspirin did not further improve the growth-inhibitory effect of 5-FU in both SW480-FU and SW620 cells with p65 knockdown (Fig. 2G,H, and Supplementary Fig. 2A). Next, we evaluated whether aspirin enhanced the invasion-inhibitory effect of 5-FU on CRC depends on the NF-kB pathway. Indeed, aspirin did not significantly improve the effect of 5-FU on the invasion of chemoresistant CRC cells with p65 knockdown by siRNA in these cells (Fig. 2I, and Supplementary Fig. 2B). These results demonstrated that aspirin enhanced growth- and invasion-inhibitory effects of 5-FU in CRC cells mainly through the NF-kB pathway.

The above results indicated that aspirin restored the sensitivity of chemoresistant CRC cells to 5-Fu, which was possibly achieved through inhibiting the 5-Fu-induced abnormal activation of NF-κB.

### Aspirin and 5-Fu synergistically inhibited tumor growth *in vivo*

**Figure 3:**
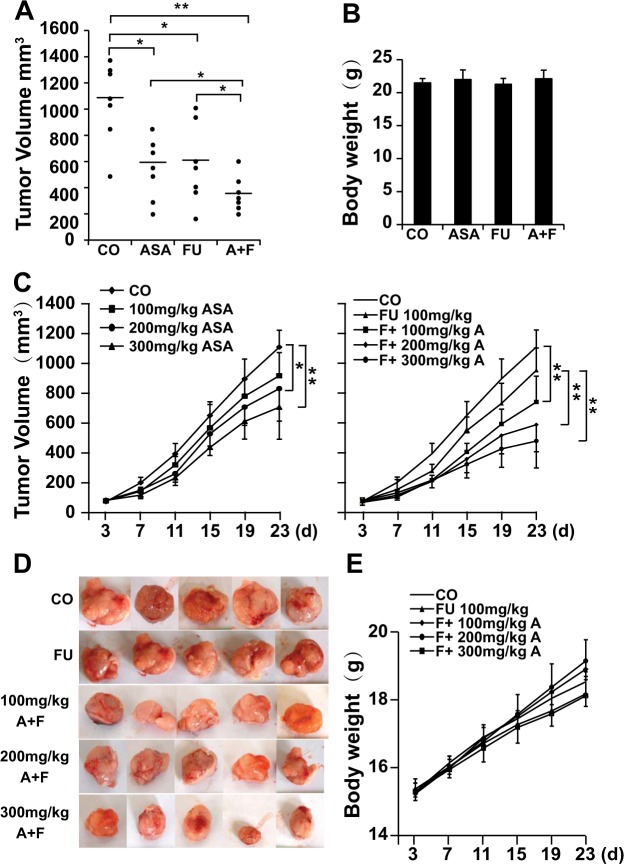
Aspirin enhances the effects of 5-Fu on suppressing tumor growth *in vivo*. **(A) S**W620 cells are injected subcutaneously into the left flanks of nude mice. 28 mice were randomized into four groups and treated with water, ASA alone (200 mg/kg, daily), FU alone (20 mg/kg, twice/week), and the combination of FU and ASA with the same dose and frequency, respectively. The xenografts are resected on day 23 and the tumor volumes were measured and analyzed. **(B)** The final body weight of mice in each group was shown. **(C)** SW620-FU cells were injected subcutaneously into the left flanks of 40 nude mice (Day 0). 40 mice were randomized into 8 groups and treated by the method described in the Materials and Methods and euthanized on day 23, and representative tumor images of each group were displayed. The tumor growth curve after treatment was analyzed and shown. **(D)** The representative photographs of tumors. **(E)** The curve of body weight growth was shown. The diagram presents the single measurements and the means ± SD (**P < 0.01, *P < 0.05).

To assess the *in vivo* effects of aspirin and 5-Fu alone or combination, both SW620 and 5-Fu-resistant SW620-FU cells were adopted in animal experiments. The final tumor volume was markedly lower in the combined treatment group than that in 5-Fu alone or control group in the tumor-bearing SW620 mice (Fig. 3A), indicating the synergistic antitumor effect between aspirin and 5-Fu *in vivo*. In addition, the final body weight of mice was not markedly changed in each treatment group compared with that of control (Fig. 3B), suggesting that mice were well tolerant to the respective treatment.

Afterwards, the effects of each treatment on the growth of SW620-FU-derived tumors were also evaluated. The results demonstrated that aspirin treatment remarkably suppressed tumor growth in a dose-dependent manner (Fig. 3C). 5-Fu treatment did not effectively suppress the growth of SW620-FU-derived tumors, even though its concentration was increased from 20 mg/kg to 25 mg/kg. However, the tumor volumes in all combined treatment groups were dramatically smaller than that in 5-Fu alone or control group (Fig. 3C,D). Importantly, the weight growth curve suggested that the weight of nude mice in the 5-Fu treatment group was slower than that in the control group, even though the difference was not statistically significant, but the combined treatment of aspirin with 5-Fu did not aggravate such trend (Fig. 3E). Moreover, the liver and kidney function indexes revealed that the plasma levels of aspartate aminotransferase (AST) and blood urea nitrogen (BUN) were not markedly elevated in the combined treatment group relative to those in 5-Fu alone group (Supplementary Fig. 3), indicating that the mice were well tolerant to the combined treatment of 5-Fu (25 mg/kg, twice a week) and aspirin (200 mg/kg, daily), without inducing additional liver and kidney toxicity.

To sum up, these data showed that aspirin had strong an antitumor effect even on 5-Fu resistant CRC cells, which enhanced the 5-Fu-induced toxicity to the resistant CRC cells without causing additional AEs *in vivo*.

### Aspirin potentiated the effect of 5-Fu on the migration, invasion, and metastasis of 5-Fu-resistant CRC cells

**Figure 4:**
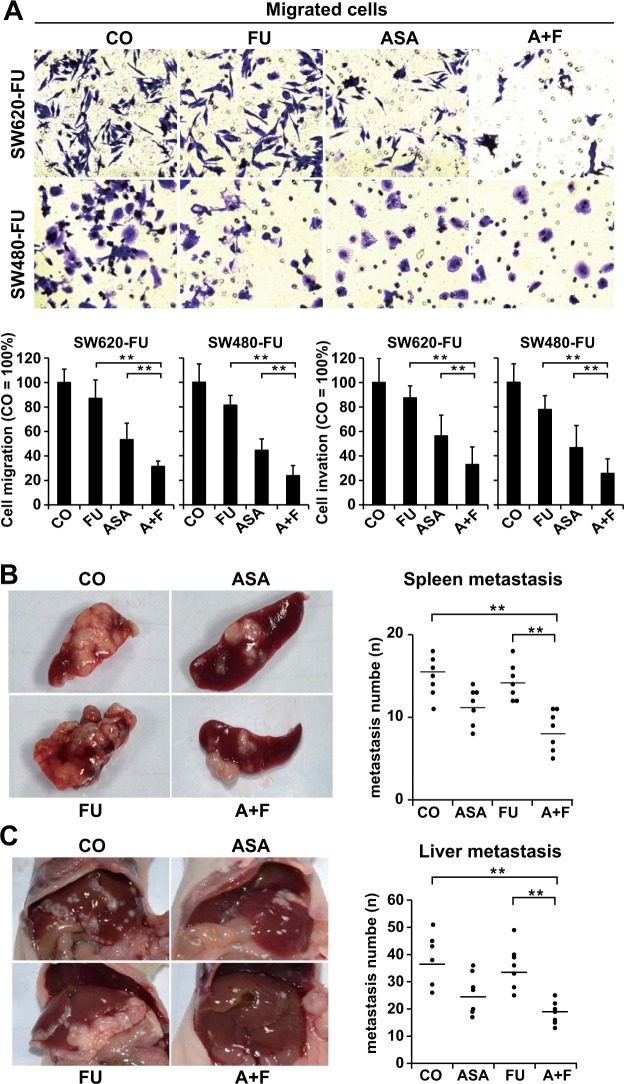
Combined application of aspirin and 5-Fu effectively inhibits the metastasis of chemoresistant CRC cells. **(A)** The invasion and migration capacities of SW620-FU and SW480-FU cells were determined using the Transwell chambers coated with or without Matrigel after 24 h of treatment with 2.5 mM ASA, 150 μM FU, or both of the two drugs (A + F). The average number of migratory cells was counted from five randomly selected fields., Representative photograph of the migrated cells was presented. The number of migratory cells in the control is set to 100%. **(B)** The spleen metastasis capacity of SW620-FU was assessed after treatment with ASA (200 mg/kg), FU (25 mg/kg), or both of the two drugs (A + F) in mice as described in the Materials and Methods. Left, representative photograph of the metastatic nodules in the spleen. **(C)** The liver metastasis capacity of SW620-FU was assessed after treatment described in the Materials and Methods. Left, representative photograph of the metastatic nodules in the liver. The number of metastatic nodules was counted and plotted. The data are presented as means ± SD (**P < 0.01, *P < 0.05).

Results of Transwell assays demonstrated that the number of migratory cells was obviously decreased after aspirin treatment, but not by 5-Fu treatment. Compared with the control group, the combined treatment group had suffered from the most significant decrease (P < 0.05) (Fig. 4A). To mimic its possible clinical setting and utility, an animal model of liver and spleen metastases had been established. In line with our *in vitro* results, it was found that aspirin treatment effectively reduced the metastatic nodes in spleen and liver compared with those in control or 5-Fu treatment group (Fig. 4B,C). Although 5-Fu treatment alone had a minimal effect on preventing the metastasis of chemoresistant CRC cells, aspirin treatment dramatically enhanced the efficacy of 5-Fu in a synergistic manner, and the greatest effect was observed in the combined treatment group. These results confirmed the antimetastatic activity of aspirin; besides, they suggested that aspirin dramatically enhanced the antimetastatic effect of 5-Fu on the chemoresistant CRC cells *in vivo*.

### Aspirin abrogated NF-κB activation stimulated by 5-Fu *in vivo*

**Figure 5:**
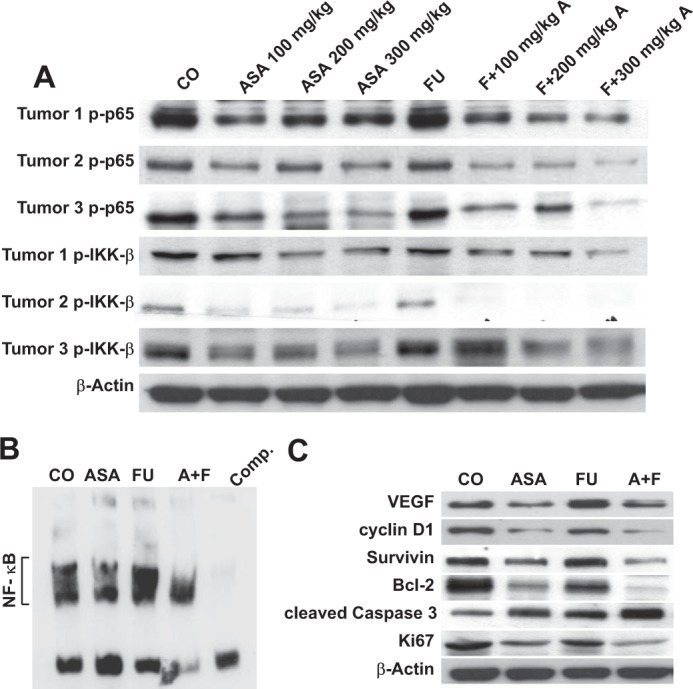
Aspirin enhances the effects of 5-FU by inhibiting NF-κB activation *in vivo*. (**A**) Western blotting analysis of p-p65 and p-IKK-β in tumor tissue samples treated with ASA alone or in combination with FU. **(B)** Detection of NF-κB by DNA binding in xenograft tumor tissues treated with ASA alone or in combination with FU. (**C**) Western blotting analysis of NF-κB-dependent gene products (VEGF, Survivin, Bcl-2, and cyclin D1) and Ki67 in colon cancer tissues treated with ASA alone or in combination with FU. Samples from three animals in each group were analyzed.

Afterwards, we investigated whether the inhibitory effect of aspirin on tumor growth in mice was associated with the suppression of NF-κB activation *in vivo*. Results of Western blotting indicated that aspirin alone evidently down-regulated the expression of p-p65 and p-IKK-β in tumor samples (Fig. 5A). More importantly, our animal studies revealed that aspirin abolished the 5-Fu-induced up-regulated expression of p-p65 and p-IKK-β Consistent with our Western blotting results, we discovered that aspirin strongly suppressed the DNA binding activity of NF-κB and attenuated the 5-Fu-induced NF-κB activation in tumor samples (Fig. 5B). Furthermore, the expression levels of downstream genes regulated by NF-κB such as Survivin, Bcl-2, VEGF, and cyclin D1, were also determined, which were found to be involved in tumor survival and chemoresistance, angiogenesis, and proliferation, respectively. Western blotting results suggested that aspirin notably down-regulated the expression levels of all these proteins, which were almost totally abolished by the combined treatment in SW620-FU tumor tissues. Moreover, Western blotting results also suggested that aspirin up-regulated the expression of pro-apoptotic factor cleaved caspase-3 while down-regulating that of the proliferative factor Ki67, and the strongest effect was observed in the combined treatment group (Fig. 5C). The above results suggested that the underlying mechanism of the synergistic antitumor effect between aspirin and 5-Fu was possibly ascribed to the fact that aspirin abolished the 5-Fu-induced NF-κB activation.

Taken together, our findings indicated that aspirin enhanced the chemotherapy sensitivity of chemoresistant CRC tumors to 5-Fu probably by suppressing the NF-κB pathway.

## Discussion

This study was the first to demonstrate that aspirin effectively re-sensitized the chemoresistant CRC cells to 5-Fu and potentiated the antitumor effects of 5-Fu on chemoresistant CRC cells without inducing additional AEs, which was achieved through suppressing the 5-Fu-induced activation of the NF-κB pathway.

Typically, chemotherapy resistance is a major cause of recurrence and metastasis after radical resection of CRC^4,26^. Although multi-drug combined therapy can partially improve the therapeutic effect, it can also lead to serious AEs^4,27^. Consequently, numerous CRC patients are forced to give up chemotherapy due to the toxicity of chemotherapeutics, which accounts for one of the leading causes of poor prognosis for CRC^27^. Our previous study suggested that aspirin evidently improved the efficacy of gemcitabine in pancreatic cancer^17^. In this study, our results showed that aspirin efficiently suppressed tumor metastasis and growth in the 5-Fu resistant CRC cells in a dose-dependent manner. Specifically, 100 mg/kg aspirin dramatically enhanced the therapeutic effect of 5-Fu, and such effect became more pronounced as the aspirin concentration increased. These results were consistent with those from previous studies showing that the administration of aspirin along with other compounds was more effective on suppressing CRC growth than either agent alone^28,29^; besides, aspirin might improve the clinical outcomes for CRC patients receiving capecitabine treatment^29^. Additionally, our study had demonstrated no cross-resistance between aspirin and 5-Fu.

5-FU at the dose of 20 mg/kg was given twice a week in this study based on a previous study^30^. Aspirin was administered at a dose of 100–200 mg/kg daily according to a previous report^17,31^ and the concentration of 2.5 mM aspirin used in our experiments was the pharmacologically relevant dose in clinical practice^32^. The dose of 100 mg/kg aspirin daily in mice is equivalent to the dose 487 mg daily for humans weighing 60 kg^17,20^. Therefore, the dose of aspirin we used for mice from 100–300 mg/kg is almost equal to about 487 to 1461 mg daily for humans, which is a dose (300–1,500 mg daily) of aspirin used in previous clinical trials^33^. High-dose aspirin (approximately 4–10 g/d) is also used clinically for the treatment of type 2 diabetic and arthritis^34,35^. Thus, aspirin was applied here in doses comparable to their clinical use. In this study, aspirin even was given at a relatively high dose (200 mg/kg), the mice still demonstrated favorable tolerability, and no additional liver and kidney toxicity was observed in the combined treatment group compared with 5-Fu alone. Such results were consistent with the clinical evidence that aspirin was very well tolerated in human subjects, even it is given at a very high dose^33^. Our results had provided an experimental basis for the clinical application of aspirin in combination with 5-Fu-based chemotherapeutics for CRC patients, especially for those with poor chemotherapy tolerance.

Epidemiological evidence and animal studies have suggested that aspirin can inhibit the liver metastasis of CRC^5,7,36^, but such effect has not been validated in the chemoresistant CRC. Therefore, the antimetastatic effect of aspirin on the chemoresistant CRC was investigated in this study, and it was found that aspirin alone had obvious antimetastatic effect, while aspirin in combination with 5-Fu had demonstrated much stronger antimetastatic effect than that of either agent alone. This was consistent with a recent clinical trial, in which the combination of aspirin and capecitabine remarkably promoted the survival of patients with metastatic CRC that failed all previous chemotherapy due to chemoresistance^37^. These results had strongly supported the synergistic antimetastatic effect between aspirin and 5-Fu in chemoresistant CRC.

The constitutive activation of NF-κB plays a crucial role in the development and chemoresistance of CRC through driving the expression of pro-proliferative/anti-apoptotic genes^38,39^, which can be further activated by chemotherapeutics^10,38,40^. In this study, we found that the basic activity of NF-κB was evidently enhanced in CRC chemoresistant cells compared with that in parental cells, indicating that NF-κB activation might account for a potential mechanism that contributed to 5-Fu resistance. This was in line with the previous studies reporting that NF-κB was enhanced in the chemoresistant cancer cells, which was responsible for the resistance of tumor cells to treatment^10,41,42^. Notably, data from our study demonstrated that aspirin almost totally abolished the 5-Fu induced NF-κB activation in chemoresistant CRC cells both *in vitro* and *in vivo*. In addition, it potentiated the effect of 5-Fu on inhibiting the NF-κB-regulated proteins, including Survivin, Bcl-2, cyclin D1, and VEGF, which were involved in cell apoptosis, proliferation, chemoresistance, and metastases in CRC^43–46^. Such results were well consistent with those studies suggesting that NF-κB was activated by the conventional chemotherapeutics, which led to chemoresistance and inactivation of the NF-κB activity, and increased the sensitivity of tumor cells to chemotherapeutics^20,40,47^. Importantly, aspirin did not significantly enhance the growth- and invasion-inhibitory effects of 5-FU on chemoresistant CRC cells knockdown of p65 by siRNA in these cells. Based on these results, we hypothesized that the aberrant activation of NF-κB induced by 5-Fu was one of the major causes leading to CRC chemoresistance and that aspirin enhanced the sensitivity of chemoresistant CRC cells to 5-Fu, which was possibly achieved through abolishing the 5-Fu-induced abnormal activation of NF-κB.

Our data had clearly suggested that aspirin monotherapy significantly suppressed tumor growth and metastasis, and its combined application with 5-Fu potentiated the antitumor effects of 5-Fu in chemoresistant CRC cells through targeting NF-κB and its downstream targets, without increasing AEs. Noteworthily, in terms of toxicity and economic costs, aspirin may serve as a very promising antitumor agent to improve the efficacy of conventional chemotherapy among CRC patients, especially for those with poor chemotherapeutic tolerance. However, further clinical studies are warranted to confirm our findings in CRC patients.

## Supplementary information

Supplementary Figure
