# Supplementary material for: Aspirin suppresses chemoresistance and enhances antitumor activity of 5-Fu in 5-Fu-resistant colorectal cancer by abolishing 5-Fu-induced NF-κB activation
Source: Sci Rep. 2019 Nov 15;9:16937. doi: 10.1038/s41598-019-53276-1 (PMC6858464; doi:10.1038/s41598-019-53276-1)
Supplement: Supplementary file 1 — Supplementary Figure [file 41598_2019_53276_MOESM1_ESM.pdf]

Aspirin suppresses chemoresistance and enhances antitumor activity of 5-Fu in 5-Fu-resistant colorectal cancer by abolishing 5-Fu induced NF-κB activation

Jinbo Fu[2,5], Yiming Xu[2,5], Yun Liu[2,5], Yushan Yang[1,3,4], Lulu Ma[1,3,4], and Yiyao Zhang [1,3,*]

[1]Departments of Gastrointestinal Surgery, Zhongshan Hospital of Xiamen University, Xiamen 361000, China
[2]General Surgery, Zhongshan Hospital of Xiamen University, Xiamen 361000, China.
[3]Gastrointestinal Oncology Center of Xiamen University, Xiamen 361000, China.
[4]Medical College of Xiamen University, Xiamen 361000, China
[5]These authors contributed equally to this work.
***Correspondence:** Yiyao zhang,Departments of Gastrointestinal Surgery, Zhongshan Hospital of Xiamen University, Xiamen 361000, China
E-mail: doctorzhyy@163.com

Supplementary Figure 1. The IC 50 values of FU in SW620 and SW480 cells.

The established CRC cell lines SW620 and SW480 were treated with indicated concentrations of FU, respectively, and the cell viability was measured at 72 h through MTT assay.

Supplementary Figure 2. Aspirin enhances the cells growth and invasion inhibitory effects of 5-FU by inhibiting NF-κB activation in vivo.

(A) SW480-FU cells were treated with 150 μM FU alone, the double combination of 2.5 mM ASA plus 150 μM FU(A+F) or 10 μM Bay11-7082 (A+B), or triple agents combination (A+B+F), respectively, and the cellular viability was measured 72 h later with an MTT assay. **(B)**The indicated cells transfected with or without p65-siRNA for 24 h were treated with 5-FU or aspirin or both agents together for 24 hours, and the invasion cells were evaluated using the Transwell chambers coated with Matrigel.

Supplementary Figure 3. Evaluation of drug toxicity to liver and kidney in nude mice.

Heparinized blood from mice was collected at the end of the experiment, and the levels of aspartate aminotransferase (AST) and blood urea nitrogen (BUN) were measured in each treatment group (200 mg/kg ASA, 25 mg/kg FU, or both of the two drugs).

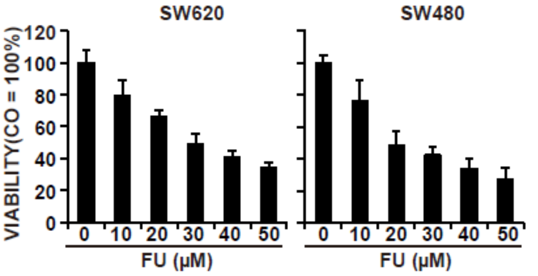

Supplementary Figure 1

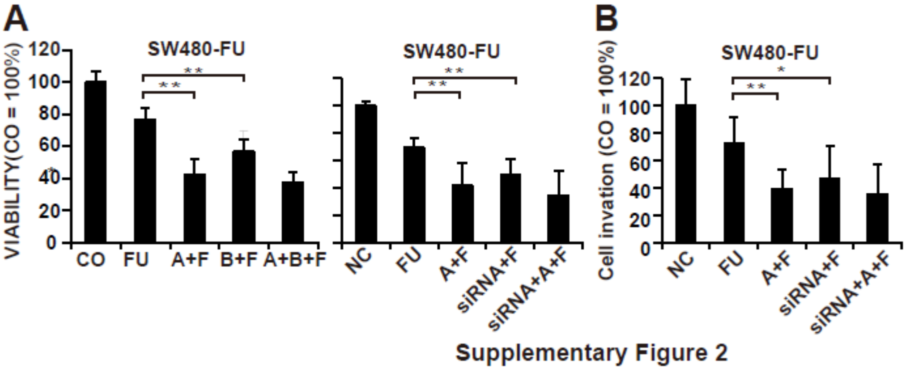

Supplementary Figure 2

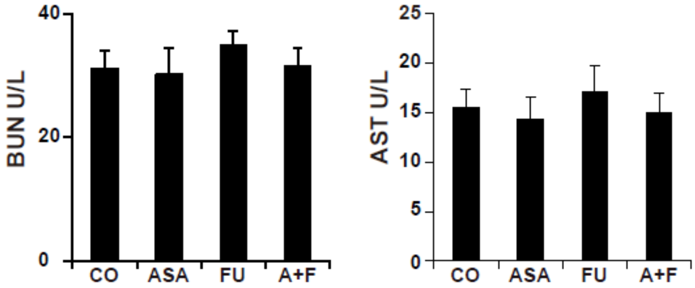

Supplementary Figure 3
